# Demographic and psychosocial risk factors for adolescent pregnancies among sexually active girls in the slums of Kampala, Uganda

**DOI:** 10.4314/ahs.v22i1.19

**Published:** 2022-03

**Authors:** Monica H Swahn, Rachel Culbreth, Sydney Adams, Rogers Kasirye, Jenelle Shanley

**Affiliations:** 1 Wellstar College of Health and Human Services, Kennesaw State University, 520 Parliament Garden Way NW, Kennesaw, GA 30144; 2 Department of Respiratory Therapy, Georgia State University, P.O. Box 4019 Atlanta, GA 30302-4019; 3 School of Public Health, Georgia State University, P.O. Box 3984 Atlanta, GA 30302-3984; 4 Uganda Youth Developmental Link P.O. Box 12659 Kampala Uganda; 5 School of Graduate Psychology, Pacific University, Hillsboro, OR 97123

## Abstract

**Background/Introduction:**

Adolescent pregnancy is a global public health issue and often linked to adverse health outcomes for both the mother and child. Youth and adolescents living in the slums of Kampala, Uganda face many environmental and psychosocial adversities, and are at a high risk of experiencing adolescent pregnancy. The goal of this study was to determine the correlates of adolescent pregnancy among sexually active girls living in the slums of Kampala.

**Methods:**

This study is based on a cross-sectional survey conducted in 2014 on youth and adolescents living in the slums of Kampala, Uganda (n=1,134) who were attending Uganda Youth Development Link drop-in centers. IRB approvals were granted.

**Results:**

In this study, 30.4% of girls reported a pregnancy. Girls who reported a pregnancy were more likely to have less than a primary education, to have lived on the streets, live in a house with more than two rooms, to drink alcohol, to have an STI, and have been raped and use condoms inconsistently, than girls who did not report a pregnancy.

**Conclusions:**

These findings may inform pregnancy prevention interventions among adolescent girls living in Kampala. Interventions may benefit from incorporating alcohol use prevention strategies, particularly for alcohol use during sex.

**Key Messages:**

- Approximately 30% of sexually active girls (ages 12–18) living in the slums of Kampala, Uganda reported a previous pregnancy.
- In the multivariable analysis, consuming alcohol during sex was the only statistically significant correlate with pregnancy.
- Intervention programs are urgently warranted to delay pregnancy and address correlates of pregnancy, such as alcohol use, for this population, as these youth and adolescents face dire environmental and psychosocial adversities.

## Introduction/background

Pregnancy among adolescents is a significant global public health issue. The impact of adolescent pregnancy on young girls is particularly significant in developing regions where 16 million girls 15–19 years old and 2.6 million girls under the age of 16 will give birth each year^1^. Moreover, the leading causes of death globally for girls 15–19 years old are complications related to pregnancy or childbearing, and adolescents under the age of 15 years old face the most significant risk of complications associated with pregnancy^1^. Adolescent mothers also face a significantly higher risk of experiencing eclampsia, puerperal endometritis, and systemic infections when compared to mothers between the ages of 20 and 24^1^–^2^.

There are many factors that are associated with an increased risk of adolescent pregnancy, including contraception choice. Some women chose to use modern forms of contraceptives such as the pill, male/female condoms, IntraUterine Device (IUD)s, implants, injectable, or traditional forms of contraceptives (e.g., herbs, rhythm methods, withdrawal). Some of the most common barriers to using modern contraceptives are cultural norms, husband or partner approval/disapproval of use, quality of services, costs associated with services, and gender-based violence^3^–^6^.

One study reported that men in the Democratic Republic of Congo perceived themselves as the sole head of the household and make all of the decisions regarding their wives' bodies and use of contraceptives^3^. Women who had more autonomy in the household were more likely to be using modern forms of contraceptives; autonomy also correlated with women's wealth. Women who were the poorest had less autonomy and were, therefore, less likely to use modern contraceptives^7^. Women were also concerned with how the community would perceive them if they used modern forms of contraceptives and these community norms influenced their decision^6^. Fear of contraceptive side effects was also a significant barrier. Some women, and men, believed that modern contraceptives could cause weight gain and infertility^3^. Money and the ability to access modern contraceptives were also factors for many women^3^, ^5^.

In addition to the complex barriers and socio-cultural norms around contraceptive use and access among adolescent girls and young women in sub-Saharan Africa, psychosocial and environmental factors have also been linked to unprotected sexual intercourse and adolescent pregnancy. Experiencing Intimate Partner Violence (IPV) is associated with barriers to contraceptive use. A study conducted by Tsai & Subramanian showed that women's condom use at last sexual intercourse was negatively associated with the appropriateness of IPV^8^. Several studies have shown that adolescents who drink alcohol are more likely to engage in condom-less sex, which may lead to sexually transmitted infection (STI) acquisition and pregnancy^9^–^10^. A dose-response relationship between alcohol use and risky sexual behaviors exist; youth who binge drink or drink excessively have a stronger association with engaging in risky sexual behaviors compared to youth who drink occasionally^11^. Alcohol consumption at the time of sexual intercourse is known to be associated with disinhibition, thus leading to more risky sexual behaviors^9^.

Parental-level factors may also be associated with risky sexual behaviors and adolescent pregnancy, including parental maltreatment of youth and parental living status. A recent meta-analysis concluded that experiencing both physical abuse and sexual abuse was strongly linked to adolescent pregnancy^12^. The association between parental living status or orphan status with adolescent pregnancy in sub-Saharan Africa has yielded mixed results in the literature. For some studies, clear associations are found with being an orphan and adolescent pregnancy, potentially due to a lack of parental oversight and guidance regarding safe sexual encounters and contraceptive methods^13^.

Environmental-level factors, such as poverty and homelessness may also impact adolescent pregnancy. Homeless adolescents in sub-Saharan Africa are more likely to engage in commercial sex work or survival sex and are also less likely to use a condom at last intercourse^14^. These adolescents have also reported a high prevalence of experiencing gender-based violence, abortions, infanticide, and child abandonment^15^.

The purpose of this project was to determine the demographic characteristics and psychosocial correlates of pregnancy among sexually active adolescent girls in the slums of Kampala, Uganda. These youth and adolescents face dire living conditions, poverty, limited food supply, and inadequate or a lack of government infrastructure as well and many health disparities noted from previous research. As such, information from our study may inform prevention and intervention programs to better support these girls and young women.

## Methods

### Setting

The current study is based on the “Kampala Youth Survey 2014”, a cross-sectional survey conducted in Spring 2014 to quantify and examine high-risk behaviors, with a focus on alcohol use, sexual behaviors and HIV. The sample consisted of urban youth and adolescents, 12–18 years of age, living in the slums or on the streets of Kampala, Uganda, who were participating in a Uganda Youth Development Link (UYDEL) drop-in center for disadvantaged street and slum youth^16^. The slums of Kampala in this study consisted of Bwaise, Kamwokya, Makindye, Nakulabye, Nateete, and Mukono.

### Data Collection

Over the 15-day data collection period, 1,628 youth and adolescents were approached for participating in the survey. Among these youth, 131 declined yielding a participation rate of 92%. A total of 1,497 surveys were collected. Three hundred and twenty (320) surveys were lost due to technical issues with the offline server, yielding 1,134 completed surveys for the final analytic sample of youth between the ages of 12 and 18 (44% boys, 56% girls).

Face-to-face interviews were conducted by social workers and peer educators employed by UYDEL with previous experience working with youth within the targeted drop-in centers and communities. Participants were informed about the study and read (or were read) the consent forms to indicate their willingness to take the survey. All participants provided verbal consent to participate in the study. Youth who “cater for their own livelihood” are considered emancipated in Uganda and are able to provide their own consent for the survey without parental consent, according to the Uganda National Council for Science and Technology board. Participation was limited to youth and adolescents ages 12–18 present in-person on the day of the field visit. IRB approvals were obtainededited out for blinded review. The Kampala Youth Survey 2014 was mostly based on previously validated quantitative measures to assess alcohol use, violence perpetration and victimization, prevalence of alcohol marketing, sexual behaviors and mental health among adolescents, including the Kampala Youth Survey 2011^17^–^21^, AUDIT Questionnaire^22^, CAGE Questionnaire^23^, and the Demographic Health Survey (USAID).

### Measures

The main outcome, ever being pregnant, was assessed using the question, “Have you or your partner ever been pregnant?” Participants could answer “Yes” or “No.” Potential confounder variables included age, education, and religion. Other environmental predictors of interest included ever living on the streets, parental living status, number of rooms in the home, time spent at UYDEL, and experiencing parental physical abuse.

Behavioral variables were also assessed, including ever using alcohol, HIV infection, STI infection, ever being raped, inconsistent condom use, alcohol use with sex, partner alcohol use with sex, sex without condom use after consuming alcohol (alcohol-related condom-less sex), regretted sex due to alcohol, multiple partners due to alcohol, age of sexual initiation, and methods used to prevent pregnancy. Participants were also asked what methods they used to prevent pregnancy, and participants could select none or as many methods as they want. Methods to prevent pregnancy included male condoms, female condoms, the pill, injectables/IUD, rhythm or withdrawal methods, and other.

Four measures on sexual norms were also assessed. Self-perceptions and norms were assessed using the question, “If I did not have sex, I would have other ways of expressing love.” Girls' perceptions of parental and adult norms were assessed using the questions, “Most adults I know discourage people my age from having sex” and “My parents would be upset if they found out that I am having sex.” Girls' perceptions of friend norms were assessed using the question, “Most of my friends think I should not have sex.” Participants could answer, “Agree,” “Neither agree nor disagree,” and “Disagree” to these four sexual norm measures.

### Data Analysis

The analyses were restricted to girls in the sample to minimize bias. Descriptive statistics were computed among all variables. Bivariate descriptive statistics among predictor variables were computed to determine differences in youth who reported pregnancy compared to youth who reported no pregnancy. Chi-square tests and Fisher Exact Tests (for expected cell counts <5) were conducted to determine differences among predictor variables in youth who reported pregnancy compared to youth who did not report pregnancy. Finally, bivariate and multivariable logistic regression analyses were conducted to determine the associations of psychosocial variables (time spent at UYDEL, ever living on the streets, parental living status, alcohol use, HIV infection, STI infection, ever being raped, parental abuse of youth, inconsistent condom use, and alcohol use during sex) and demographic variables (religion and education) with the outcome of ever being pregnant. Unadjusted odds ratios and adjusted odds ratios with corresponding 95% confidence intervals are presented. All analyses were conducted in SAS 9.4 (SAS Institute, Cary, NC).

### Patient and Public Involvement

Peer educators at UYDEL administered the survey, who work with the adolescents on a daily basis. UYDEL youth and adolescents assisted in recruitment strategies for participants. The design, development, and survey questionnaire was constructed with UYDEL staff who know the girls' needs. Additionally, these results will be used to directly inform UYDEL interventions and initiatives to benefit youth at UYDEL drop-in centers.

## Results

In this study, 30.4% of girls ages 12–18 (n=349) reported a pregnancy (Tables 1). Girls who reported a pregnancy were more likely to have less than a primary education, to have lived on the streets, live in a house with more than two rooms, to drink alcohol, to have an STI, and have been raped and use condoms inconsistently, than girls who did not report a pregnancy. Moreover, girls who reported a pregnancy were also more likely to report drinking alcohol in general, drinking alcohol at time of sex, having a partner drinking at time of sex, not using a condom because of alcohol at time of sex, regretting sex due to alcohol, and having multiple partners due to alcohol than girls who did not report a pregnancy. There were no differences between girls who reported a pregnancy and those who did not with respect to age of sexual initiation, religious affiliation, adult, parental and friend norms around sexual activity or whether they could find other ways to express love rather than having sex. In multivariable analyses (Table 2), the only significant correlate of pregnancy was alcohol use at time of sex (Adj.OR:3.63; 95% CI: 1.67–7.89).

**Table 1:** Demographic and Psychosocial Characteristics of Pregnancy among sexually active girls 12–18 years of age in Kampala (n=349)

	Pregnancy Yes N=106 (30.4%)	Pregnancy No N=243 (69.6%)	Total n= 349 (100%)	Chi-Square, df, p-value
Age, M (SD)*	17.2 (1.0)	16.7 (1.4)	16.9 (1.3)	-3.95, (271), p<0.0001
Education Less than primary Completed primary Secondary or higher	42 (40.0%) 18 (17.1%) 45 (42.9%)	79 (32.9%) 56 (23.3%) 105 (43.8%)	121 (35.1%) 74 (21.5%) 150 (43.5%)	2.36, (2), p=0.31
Ever lived on streets Yes No	39 (36.8%) 67 (63.2%)	49 (20.2%) 194 (79.8%)	88 (25.2%) 261 (75.8%)	10.82, (1), p=0.001
Parental living status Both parents living One parent dead Both parents dead	30 (28.3%) 43 (40.6%) 33 (31.1%)	77 (31.7%) 104 (42.8%) 62 (25.5%)	107 (30.7%) 147 (42.1%) 95 (27.2%)	1.22, (2), p=0.54
Rooms in the house** One Two More than two Other/other arrangement	53 (34.4%) 28 (26.4%) 23 (39.0%) 2 (1.9%)	101 (41.6%) 101 (41.6%) 36 (14.8%) 5 (2.1%)	154 (44%) 129 (40.0%) 59 (16.9%) 7 (2.0%)	p=0.04
Religion Christian- Catholic Christian- other Muslim Other	43 (50.6%) 34 (32.1%) 21 (19.8%) 8 (7.6%)	89 (36.6%) 101 (41.6%) 41 (16.9%) 12 (4.9%)	132 (37.8%) 135 (38.7%) 62 (17.8%) 20 (5.7%)	3.26, (3), p=0.35
UYDEL affiliation Less than 1 week 1–4 weeks 1 month to 1 year 1 year or longer	34 (34%) 30 (30.3%) 31 (31.3%) 4 (4.0%)	73 (33.5%) 50 (22.9%) 86 (39.5%) 9 (4.1%)	107 (33.8%) 80 (25.2%) 117 (36.9%) 13 (4.1%)	2.70, (3), p=0.44
Alcohol use Yes No	68 (64.2%) 38 (35.9%)	106 (43.6%) 137 (56.4%)	174 (49.9%) 175 (50.1%)	12.44, (1), p=0.0004
HIV infection Yes No	19 (17.9%) 87 (82.1%)	31 (12.9%) 209 (87.1%)	50 (14.5%) 296 (85.6%)	1.49, (1), p=0.22
STI Infection Yes No	73 (68.9%) 33 (31.1%)	135 (55.6%) 108 (44.4%)	208 (59.6%) 141 (40.4%)	5.43, (1), p=0.02
Ever been raped Yes No	48 (45.3%) 58 (54.7%)	80 (32.9%) 163 (67.1%)	128 (35.7%) 221 (63.3%)	4.86, (1), 0.03
Inconsistent condom use Yes No	75 (79.8%) 19 (20.2%)	143 (66.8%) 71 (33.2%)	218 (70.8%) 90 (29.2%)	5.31, (1), p=0.02
Alcohol use with sex Yes No	48 (45.3%) 58 (54.7%)	44 (18.2%) 198 (81.8%)	92 (26.4%) 256 (73.6%)	27.84, (1), p<0.0001
Partner alcohol use with sex Yes No	49 (46.2%) 57 (53.8%)	47 (19.4%0 195 (80.6%)	96 (27.6%) 252 (72.4%)	26.51, (1), p<0.0001
Alcohol-related condom-less sex Yes No	48 (45.3%) 58 (54.7%)	61 (25.1%) 182 (74.9%)	109 (31.2%) 240 (68.8%)	13.99, (1), p=0.0002
Regretted sex due to alcohol Yes No	46 (43.4%) 60 (56.6%)	59 (24.3%) 184 (75.7%)	105 (30.1%) 244 (69.9%)	12.82, (1), p=0.0003
Multiple partners due to alcohol Yes No	31 (29.3%) 75 (70.8%)	37 (15.2%) 206 (84.8%)	68 (19.5%) 281 (80.5%)	9.25, (1), p=0.002
If I did not have sex, I would have other ways of expressing love Agree Neither agree nor disagree Disagree	79 (74.5%) 4 (3.8%) 23 (21.7%)	182 (75.2%) 25 (10.3%) 35 (14.5%)	261 (75.0%) 29 (8.3%) 58 (16.7%)	6.12, (2), p=0.05
Most of my friends think I should not have sex Agree Neither agree nor disagree Disagree	35 (33.0%) 28 (26.4%) 43 (40.6%)	105 (43.4%) 61 (25.2%) 76 (31.4%)	140 (40.2%) 89 (25.6%) 119 (34.2%)	3.82, (2), p=0.15
Most adults I know discourage people my age from having sex Agree Neither agree nor disagree Disagree	83 (78.3%) 8 (7.6%) 15 (14.2%)	182 (74.9%) 26 (10.7%) 35 (14.4%)	265 (75.9%) 34 (9.7%) 50 (14.3%)	0.87, (2), p=0.65
My parents would be upset if they found out that I am having sex Agree Neither agree nor disagree Disagree	74 (69.8%) 21 (19.8%) 11 (10.4%)	190 (78.2%) 31 (12.8%) 22 (9.1%)	264 (75.6%) 52 (14.9%) 33 (9.5%)	3.29, (2), p=0.19
Age of sexual initiation < 12 13–14 15–16 17–18	12 (11.3%) 41 (38.7%) 36 (34.0%) 17 (16.0%)	28 (11.5%) 71 (29.2%) 99 (40.7%) 45 (18.5%)	40 (11.5%) 112 (32.1%) 135 (38.7%) 62 (17.8%	
Method used to prevent pregnancy*** Male condom Female condom The pill Injectables, Depo, IUD Rhythm/withdrawal Other	80 (75.5%) 9 (8.5%) 31 (29.2%) 8 (7.5%) 15 (14.2%) 21 (19.8%)	201 (82.7%) 9 (3.7%) 28 (11.5%) 10 (4.1%) 28 (11.5%) 7 (2.9%)	281 (80.5%) 18 (5.2%) 59 (16.9%) 18 (5.2%) 43 (17.7%) 28 (8.0%)	N/A
Parental physical abuse of youth Yes No	43 (40.6%) 63 (59.4%)	94 (38.8%) 148 (61.2%)	137 (39.4%) 211 (60.6%)	0.09, (1), p=0.76

* T-test used for continuous variable age.

** missing 50% of data

*** Not mutually exclusive categories; therefore, chi-squares not computed.

**** Fisher exact test used because expected cell sizes <5.

**Table 2:** Associations between psychosocial risk factors and pregnancy among Demographic and Psychosocial Characteristics of Pregnancy among girls 12–18 years of age in Kampala (n=349)

	Unadjusted OR (95% CI)	Adjusted OR (95% CI)
Religion Christian- Catholic Christian- other Muslim Other	1.00 0.70 (0.41, 1.19) 1.06 (0.56, 2.01) 1.38 (0.53, 3.63)	1.00 0.63 (0.32, 1.22) 1.52 (0.68, 3.42) 0.89 (0.26, 2.97)
Time spent at UYDEL <1 week 1–4 weeks 1 month to 1 year 1 year or longer	1.00 1.29 (0.70, 2.37) 0.77 (0.43, 1.38) 0.95 (0.27, 3.32)	1.00 1.09 (0.52, 2.30) 0.58 (0.13, 2.52) 0.64 (0.28, 1.43)
Education Less than primary Completed primary Secondary or higher	1.00 0.61 (0.32, 1.16) 0.81 (0.48, 1.35)	1.00 0.64 (0.28, 1.43) 1.22 (0.65, 2.32)
Ever lived on streets Yes No	**2.31 (1.39, 3.82)** 1.00	0.85 (0.41, 1.73) 1.00
Parental living status Both parents living One parent dead Both parents dead	1.00 1.06 (0.61, 1.84) 1.37 (0.75, 2.48)	1.00 0.97 (0.49, 1.92) 1.27 (0.59, 2.73)
Alcohol use Yes No	**2.31 (1.44, 3.70)** 1.00	1.55 (0.77, 3.13) 1.00
HIV infection Yes No	1.47 (0.80, 2.75) 1.00	1.13 (0.49, 2.57) 1.00
STI Infection Yes No	**1.77 (1.09, 2.87)** 1.00	0.92 (0.48, 1.78) 1.00
Ever been raped Yes No	**1.69 (1.06, 2.69)** 1.00	1.69 (0.92, 3.10) 1.00
Parental abuse of child Yes No	1.08 (0.67, 1.71) 1.00	1.01 (0.54, 1.87) 1.00
Inconsistent condom use Yes No	**1.96 (1.10, 3.49)** 1.00	1.78 (0.93, 3.41) 1.00
Alcohol during sex Yes No	**3.72 (2.25, 6.16)** 1.00	**3.63 (1.67, 7.89)** 1.00

## Conclusion

In this study we examined the prevalence and correlates of pregnancy among girls living in the slums of Kampala, Uganda. In this young sample of sexually active girls, ages 12 to 18 years, 30 percent had already been pregnant. This is higher than the 25% of teenagers who become pregnant by age 19 in Uganda, according to the Ministry of Health^25^.

Our analyses show that several factors pertaining to hardship and disparities such as living on the street, not completing education, increased the likelihood of a pregnancy. However, in terms of behavioral risk factors, several important findings were noted. Girls who reported a pregnancy were more likely to drink alcohol overall and more likely to drink alcohol at time of sex. In fact, in the multivariable analyses, drinking alcohol at time of sex was the only single statistically significant factor associated with pregnancy.

While there is extensive research on alcohol use and its potential harm in terms of sexually transmitted infections, including HIV, and other harms such as violence and injury, few studies have examined the role of alcohol in adolescent pregnancy, which is another burden for many young girls not planning to become pregnant, especially while attending school.

Adolescent pregnancy prevention mainly targets literacy and education, but it is unclear whether any teenage pregnancy prevention programs address alcohol use and drinking at time of sex specifically. It is well documented that youth and adolescents are less likely to use a condom while drinking^26^–^27^. Moreover, girls often report difficulty in condom use negotiation, particularly when violence is experienced^28^. It is likely that drinking alcohol serves as a key barrier to negotiating condom use for girls^29^.

Our study did not find any differences with respect to self-efficacy measures or norms surrounding sexual activity. Moreover, reporting pregnancy did not vary based on the age of sexual debut or by religious affiliation. Perhaps most noteworthy, is our findings that the norms or opinions by other adults, by their parents or friends with respect to sexual activity did not differ for girls who reported a pregnancy compared to those who did not. This is very intriguing and troubling, as early pregnancy prevention campaigns seek to create social norms that disapprove of sexual activity. It may be that the more proximal factors, such as alcohol use at time of sex, is a much more powerful risk factor for adolescent pregnancy and will need a different intervention strategy.

Several limitations should be considered when interpreting the findings from this study. First, our findings are based on a convenience sample of service-seeking girls. However, these girls are often hard to reach, and a convenience sample may be one of the few strategies to sample these girls. Additionally, the adolescents in this study were seeking services at UYDEL, which may limit generalizability to other service-seeking adolescents living in the slums or in similar settings. Recall bias and social desirability bias may also impact misclassification of adolescents in this study, specifically among sensitive questions regarding pregnancy, sexual activity and alcohol use. Moreover, the study was not conducted specifically to determine pregnancy and related outcomes as such there is very limited information available to provide additional context about the status of the pregnancy, how many pregnancies there had been and whether any of the pregnancies resulted in the birth of a child. Finally, our sample of sexually active girls was relatively small (N=349) which may have impacted the strength of the statistically findings observed.

Despite these limitations, this study is the first study to our knowledge, to examine the prevalence and correlates of pregnancy among vulnerable girls in the slums of Kampala and in the broader region. Clearly, with 30 percent of the girls reporting a pregnancy this is an urgent priority for prevention. Our findings show that alcohol use, particularly at the time of sex, is a very strong risk factor for pregnancy in this population, which suggest that alcohol use prevention strategies need to be included in broader pregnancy prevention initiatives among vulnerable girls in the slums.

In Uganda, policies targeted towards adolescence and reproductive services are favorable; however, these policies are not reflected in the majority of health clinics across Uganda 30. Only 10% of the health clinics cater to adolescent healthcare needs, while policymakers estimate that Uganda needs 75% of all health clinics to offer adolescent services^31^. Recommendations for the prevention of adolescent pregnancy in Uganda also include the incorporation of reproductive health education in school programs, the accessibility of educational information for adolescence, and an increase in the number of health facilities with access for adolescents^30^.
